# A case of spontaneous mesenteric hematoma successfully diagnosed and treated with aggressive imaging

**DOI:** 10.1016/j.ijscr.2019.10.058

**Published:** 2019-10-31

**Authors:** Shunsuke Nakamura, Taihei Yamada, Tsuyoshi Nojima, Hiromichi Naito, Hitoshi Koga, Hisashi Yamashita, Akira Gochi, Atsunori Nakao

**Affiliations:** a Department of Emergency, Critical Care and Disaster Medicine, Okayama University Graduate School of Medicine Dentistry and Pharmaceutical Sciences, Japan; b Department of Emergency Medicine, St. Mary’s Hospital, Kurume, Fukuoka, Japan; c Department of Surgery, Ibara City Hospital, Okayama, Japan; d Center for Graduate Medical Education, Okayama University Hospital, Japan

**Keywords:** Mesenteric hematoma, Computed tomography, Acute care surgery

## Abstract

**Introduction:**

Spontaneous mesenteric hematoma is an uncommon syndrome triggered by bleeding localized in the mesenteric vascular tree of a bowel segment for no apparent underlying reason. We herein report a surgical patient with an extremely rapidly growing spontaneous mesenteric hematoma that we successfully diagnosed using careful radiologic examination.

**Presentation of case:**

A 56-year-old old male presenting sudden onset lower abdominal pain was referred to our emergency department. At the time of admission, his physical examination revealed stable vital signs without radiological abnormality. On the following day, the patient suddenly presented hypotension, tachycardia, and increased abdominal pain. Contrast-enhanced computed tomography examination showed a mass with both high- and low-density areas with a 130 mm maximum diameter bordering the transverse colon. Since interventional radiologists were not available, we decided to perform emergency exploratory laparotomy. On laparotomy, a 13 × 8 cm hematoma was found in the mesentery of the transverse colon. As bleeding was noted from the branches of the middle colic artery and gastrocolic artery, these responsible vessels were ligated. The patient was finally given the diagnosis of spontaneous mesenteric hematoma.

**Discussion and conclusion:**

The present case, initially diagnosed as enterocolitis, suddenly manifested hypovolemic shock. Close monitoring for any signs of further deterioration, as well as aggressive imaging diagnosis, enabled us to avoid delays in treatment. Early diagnosis and treatment of mesenteric hematomas are essential to prevent them from rupturing and triggering life-threatening adverse events.

## Highlights

• Spontaneous mesenteric hematoma is an uncommon syndrome, and often misdiagnosed as other non-hemorrhagic acute abdomen.
• Close monitoring for any signs of further deterioration, as well as aggressive imaging diagnosis, enabled us to make early diagnosis and treatment.
• Sharing our experience may help physicians initiate treatment of mesenteric hematomas early to prevent life-threatening adverse events.

## 1 Introduction

Mesenteric hematoma is an uncommon syndrome caused by localized bleeding in the mesenteric vascular tree. Mesenteric hematomas are often caused by trauma or abdominal surgeries [1,2]. Otherwise, mesenteric hematomas are usually associated with coagulopathies [3], connective tissue disorders [4], arteriopathy [5], pancreatitis [6], or bleeding from visceral artery aneurysms [7]. Spontaneous (or idiopathic) mesenteric hematoma is suspected when pathological and clinical causes are not found during progression of the condition [8]. We herein report a surgical patient with an extremely rapidly growing spontaneous mesenteric hematoma that we successfully diagnosed using careful radiologic examination. Sharing our experience may help emergency physicians diagnose and initiate treatment of mesenteric hematomas early to prevent rupture and subsequent life-threatening adverse events.

## 2 Presentation of case

A 56-year-old old male presenting sudden onset lower abdominal pain was referred to our emergency department. He had been well, was taking no medication, and had no allergies. At the time of admission, his physical examination revealed stable vital signs and tenderness in the lower abdomen without peritoneal irritation.

His laboratory test results were unremarkable besides mild leukocytosis with a prothrombin time of 11.1 s. Since his radiological exam results were unremarkable without ascites, bowel obstruction, or pneumoperitoneum, the patient was diagnosed with acute enterocolitis and admitted to our department for careful observation.

On the following day, the patient complained of intermittent severe abdominal pain. Plain computed tomography (CT) examination was unremarkable. However, one hour after a second CT examination, the patient presented hypotension, tachycardia, and increased abdominal pain with muscle defense and a palpable abdominal mass. Suspecting aortic aneurysm rupture, we again performed a contrast-enhanced CT examination with rapid fluid infusion, which showed a mass with both high- and low-density areas with a 13 cm maximum diameter bordering the transverse colon. Blood cell counts demonstrated a decrease of hemoglobin level from 18 g/dL at presentation to 10 g/dL before the second CT evaluation.

**Fig. 1:**
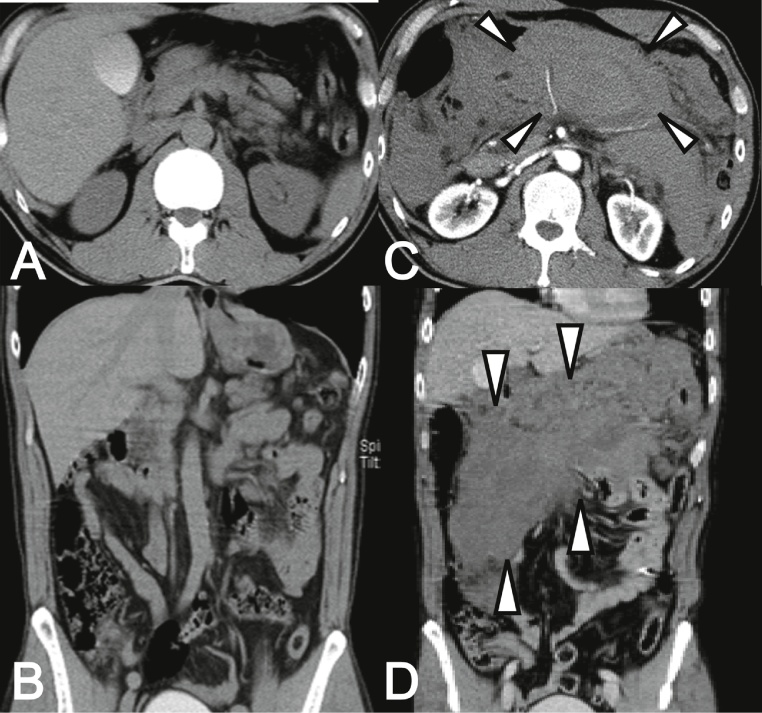
Plain computed tomography (CT) examination on day 2 was unremarkable (A, B). Contrast-enhanced CT examination with rapid fluid infusion, performed immediately after resuscitation from hypovolemic shock, revealed a mass with both high- and low-density areas with a 13 cm maximum diameter bordering the transverse colon (arrowhead, C, D).

Since interventional radiologists were not available, we decided to perform emergency exploratory laparotomy. On laparotomy, a 13 × 8 cm hematoma was found located in the mesentery of the transverse colon. As bleeding was noted from the branches of the middle colic artery and gastrocolic artery, these responsible vessels were ligated. No bloody ascites had accumulated in the abdominal cavity. The serosa of the intestine and colon appeared normal. The patient’s postoperative course was unremarkable, and he was discharged on the 14th hospital day. The patient has been well without recurrence of abdominal pain. Pathological and surgical findings did not reveal the reason for the bleeding. The patient was finally diagnosed with spontaneous mesenteric hematoma since he had no medical history associated with development of a mesenteric hematoma such as anticoagulant treatment, labor, trauma, or surgery (Fig. 1).

## 3 Discussion

Idiopathic mesenteric hematoma is an extremely rare disease characterized by sudden bleeding into the mesentery without any cause, making it difficult to diagnose. The condition presents non-specific symptoms with various onsets, including diarrhea, vomiting, abdominal pain, abdominal mass, and melena. Accelerating bleeding can induce hypotension and hypovolemic shock. Emergency physicians need to distinguish mesenteric hematoma from mesenteric ischemia or intraperitoneal hemorrhage.

Diagnosing mesenteric hematoma before a patient develops hypotension is challenging, but critical. CT scanning is the standard imaging modality used to identify mesenteric hematoma. CT is required to rule out other more common causes of abdominal pain and shock, including abdominal aneurysm, malignancy, and acute pancreatitis. If the patient is hemodynamically stable and contrast-enhanced CT suggests a diagnosis of mesenteric hematoma for the bleeding origin, selective visceral angiography should be performed and the bleeding vessels should be embolized if identified [9].

Management of mesenteric hematoma remains controversial and depends on the patient’s clinical stability. The condition can be treated with non-surgical, conservative techniques if the hemorrhage is controlled without active bleeding. On the other hand, hypotensive patients that don’t respond to fluid resuscitation need emergency operation or emergency interventional arterial embolization. A laparoscopic approach rather than laparotomy may be highly effective for patients in relatively stable condition due to many advantages such as reduced respiratory complications, reduced wound pain, and better visibility of the abdominal cavity [10].

The present case, initially diagnosed as clinically stable enterocolitis, suddenly manifested hypovolemic shock. Close monitoring for any signs of further deterioration, as well as aggressive urgent imaging diagnosis after resuscitation, enabled us to avoid delays in treatment. Prolonged prothrombin time (PT) might help emergency physicians make decisions when developing a therapeutic strategy [11]. The PT in our case was 11.1 s (PT-INR 0.95) at presentation and 14.2 s (PT-INR 1.22) before the last CT evaluation, respectively. Bleeding vessels were not identified in around 40% of mesenteric hematomas in previous case series reports [12]. We identified and ligated the bleeding vessels; however, even retrospective review of the CT angiography images failed to reveal any pathologies in the vessels prior to bleeding.

We believe that emergency interventional arterial embolization may be the best strategy if the bleeding vessels can be identified. However, as in our center, radiologists are not always available in some hospitals. Transferring a patient in hypovolemic shock may present a high risk. Sharing our experience may help emergency physicians devise therapeutic strategies for similar cases.

## 4 Conclusion

Spontaneous mesenteric hematoma is an uncommon syndrome triggered by localized bleeding in the mesenteric vascular tree of a bowel segment for no apparent underlying reason. Delay in diagnosis may be fatal, and when this disease is suspected, it is necessary to perform aggressive imaging to rule out differential diagnoses. Patients should be monitored closely for any signs of further deterioration.

## Declaration

We declare that our case is compliant with the SCARE criteria [13].

## Sources of funding

None.

## Ethical approval

This case study was approved by the ethical committee of Okayama University.

## Consent

Fully informed written consent was obtained.

## Author contribution

Shunsuke Nakamura, Taihei Yamada, Hiromichi Naito, Tsuyoshi Nojima, and Atsunori Nakao treated the patient and collected data. Hitoshi Koga, Hisashi Yamashita and Akira Gochi contributed to the study design, data analysis, and review Hiromichi Naito and Atsunori Nakao wrote the paper.

## Registration of research studies

Not applicable

## Guarantor

Hiromichi Naito.

## Provenance and peer review

Not commissioned, externally peer-reviewed

## Declaration of Competing Interest

None.
